# A special case of intrahepatic cholangiocarcinoma misdiagnosed as hepatic cystic echinococcosis

**DOI:** 10.1016/j.heliyon.2024.e35073

**Published:** 2024-07-23

**Authors:** Dalong Zhu, Abuduhaiwaier Abuduhelili, Alimu Tulahong, Chang Liu, Tiemin Jiang, Yingmei Shao, Tuerganaili Aji

**Affiliations:** a Department of Hepatobiliary and Echinococcosis Surgery, Digestive and Vascular Surgery Center, The First Affiliated Hospital of Xinjiang Medical University, Urumqi, 830054, Xinjiang Uygur Autonomous Region, China; b Xinjiang Uyghur Autonomous Region Clinical Research Center for Echinococcosis and Hepatobiliary Diseases, The First Affiliated Hospital of Xinjiang Medical University, Urumqi, 830054, Xinjiang Uygur Autonomous Region, China; c The First Affiliated Hospital of Xinjiang Medical University, Urumqi, 830054, Xinjiang Uygur Autonomous Region, China

**Keywords:** Intrahepatic cholangiocarcinoma, Hepatic cystic echinococcosis, Necrosis, Misdiagnosis, Case report

## Abstract

Intrahepatic cholangiocarcinoma (iCCA) is a prevalent liver tumor that presents a diagnostic challenge due to its nonspecific symptoms, necessitating reliance on imaging techniques for accurate diagnosis. The similarity of imaging features with other liver diseases, such as hepatocellular carcinoma (HCC) and hepatic alveolar echinococcosis, often leads to confusion and misdiagnosis. In contrast, the distinct characteristics of hepatic cystic echinococcosis (HCE) result in fewer reported misdiagnoses. A case involving a 53-year-old female from Changji (Xinjiang, China) diagnosed with iCCA, who was hospitalized for symptoms of upper abdominal distension and pain, along with nausea and vomiting, is presented. The patient underwent a partial hepatectomy in 1990 for hepatic echinococcosis. Abdominal computed tomography revealed multiple, quasicircular, low-density masses in the hilar region and right anterior lobe of the liver, with the largest measuring 5.61 cm × 4.84 cm. Enhanced computed tomography did not reveal significant enhancement of the lesion. Considering epidemiological factors, medical history, and imaging findings, the initial diagnosis was HCE, which prompted surgical intervention. The diagnosis of iCCA with necrosis was confirmed via pathological examination. The literature and relevant sources were consulted to establish that biliary tract tumors with necrosis or mucin production typically do not exhibit significant enhancement in enhanced scans, maintaining a consistently low density across all phases, resembling the presentation of HCE. When making diagnoses based on imaging data, it is essential to have knowledge of both the typical features and unique manifestations of the disease. In specific instances, relying solely on epidemiology and medical history may lead to incorrect conclusions. Therefore, comprehensive consideration of all aspects is necessary to prevent missed diagnoses and misdiagnoses.

## 1 Introduction

Cholangiocarcinoma (CCA) is a malignant neoplasm originating from epithelial cells lining the bile ducts and adjacent glands, and is the second most prevalent liver tumor, trailing only hepatocellular carcinoma (HCC) [[1], [2], [3], [4]]. Anatomically, CCA can be categorized into intrahepatic CCA (iCCA), distal CCA, and perihilar CCA subtypes, with iCCA representing the least common variant, accounting for approximately 10 % of cases. iCCA frequently presents with nonspecific early symptoms and is commonly detected incidentally as a liver mass on imaging studies, posing a diagnostic challenge due to potential confusion with HCC, metastatic adenocarcinoma, or hepatic alveolar echinococcosis [3,5,6]. Hepatic cystic echinococcosis (HCE) results from infection with *Echinococcus granulosus* and manifests as cystic lesions that typically exhibit a round shape and fluid-like density. Instances of iCCA and HCE misdiagnosis have been infrequently documented in the literature. Herein, we present a case of a hepatic tumor with cystic lesions that was diagnosed as HCE after thorough consideration of epidemiological, historical, and imaging data. Following surgical resection of the lesion, it was pathologically confirmed to be iCCA with necrosis. We hope that this case of misdiagnosis can serve as a differential diagnostic reference for other clinicians when they are diagnosing similar cases.

## 2 Case presentation

A 53-year-old female of Han Chinese descent from Changji (Xinjiang, China) was admitted to the authors' hospital with complaints of epigastric pain, nausea, and vomiting that had recurred in the past month. The patient reported no history of chronic illnesses but disclosed an allergy to penicillin. She underwent partial hepatectomy for hepatic echinococcosis in 1990. On admission, the patient exhibited stable vital signs, abdominal tenderness, and no rebound tenderness. A linear scar from a previous surgical procedure, measuring approximately 10 cm in length, was observed in the upper abdominal region; the remainder of the physical examination was unremarkable. The patient underwent a series of laboratory and imaging investigations, yielding the following findings: total bilirubin, 40.59 μmoL/l; serum aspartate aminotransferase, 100.05 U/L; serum alanine aminotransferase, 120.00 U/L; serum γ-glutamyl transpeptidase, 961.78 U/L; alkaline phosphatase, 544.63 U/L; serum potassium, 2.55 mmol/L; carbohydrate antigen 19-9 (CA19-9), >1200 U/mL; carbohydrate antigen 50 (CA50), >180 U/mL; and serum ferritin, 320.89 ng/mL. All other laboratory investigation results were within the established reference ranges. Ultrasound (US) identified multiple round areas of mixed echogenicity near the second porta hepatis, characterized by poorly defined borders and heterogeneous internal changes, along with a membranous medium-level echo (Fig. 1A). Computed tomography (CT) imaging revealed several round, low-density masses located in the hepatic hilum and the anterior right lobe of the liver, with the largest measuring 5.61 cm × 4.84 cm. The internal density of the lesion was consistent, and no significant enhancement was observed following contrast administration. However, during the portal venous phase of the enhanced scan, a slightly low-density patch was identified in the left medial lobe (Fig. 1B and C). Magnetic resonance imaging (MRI) revealed round cystic lesions with prolonged T1 and T2 signals in the left hepatic hilum and right lobe, accompanied by internal strip-like signals with slightly shorter T1 and T2. The lesions measured approximately 8.19 cm × 5.25 cm and were surrounded by a ring of short T2 signals (Fig. 1D and E). After evaluation of the patient's symptoms, medical history, and laboratory and imaging results, an initial diagnosis of HCE was established. Subsequently, the patient underwent a left hemi-hepatectomy, hepaticojejunostomy, and cholecystectomy. Following the surgical procedure, specimens of the left hepatic tissue and residual cavity tissue of the liver lesion were subjected to pathological analysis, which revealed iCCA with necrosis (Fig. 1F). Postoperatively, the patient experienced intra-abdominal fluid accumulation and bile leakage, which were successfully managed with appropriate intervention(s), resulting in the absence of severe complications on discharge. During our follow-up, we learned that the patient died of severe gastrointestinal bleeding in the local hospital more than 2 months after discharge from the author's hospital (Fig. 2).

**Fig. 1:**
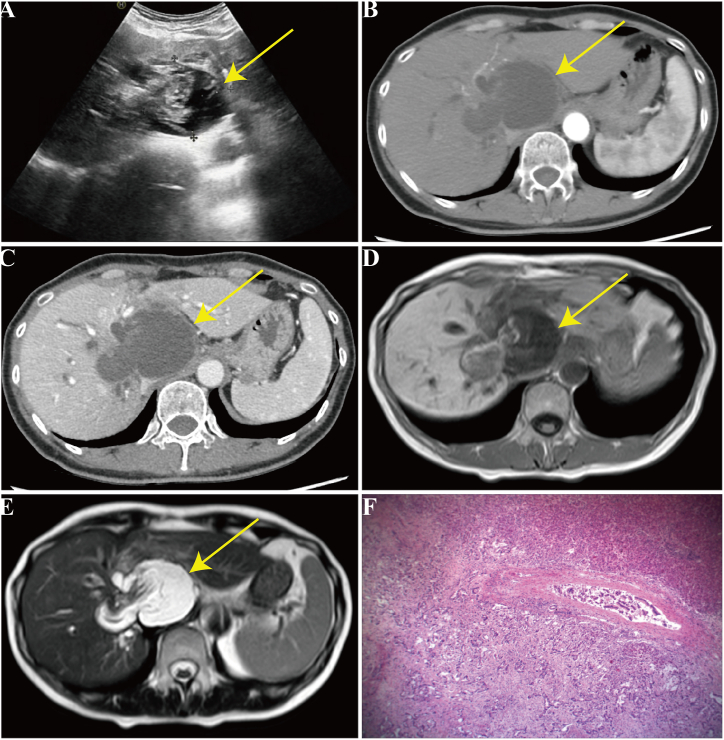
(A) Ultrasonography identified an ovoid region with mixed echogenicity, measuring approximately 6.0 cm × 5.4 cm, in close proximity to the second porta hepatis. The boundaries were poorly defined, and there was a discernible aggregation of membranous medium-level echoes present. (B,C) Computed tomography (CT) imaging demonstrating an ovoid hypodense mass, measuring 5.61 cm × 4.84 cm, at the hepatic hilum. The internal density appears uniform, and no significant enhancement was noted during either the arterial or delayed phase of the contrast-enhanced CT scan. (D,E) Magnetic resonance imaging revealing an ovoid cystic lesion adjacent to the hepatic hilum, measuring approximately 8.19 cm × 5.25 cm, exhibiting prolonged T1 and T2 signals. Within the lesion, strip-like regions with marginally shorter T1 and T2 signals are observed, along with a perimeter of short T2 signals surrounding the lesion. (F) Pathological examination confirmed moderately to poorly differentiated intrahepatic cholangiocellular carcinoma with necrosis and invasion of the outer layer of the biliary tract (hematoxylin and eosin staining, original magnification × 100).

**Fig. 2:**
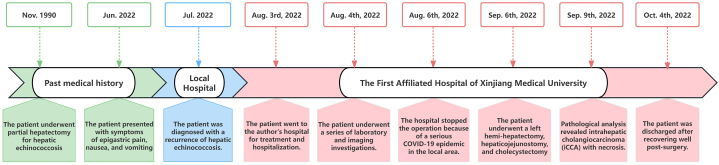
Timeline of the patient's disease occurrence, treatment, examinations, and surgery-related data.

## 3 Discussion

iCCA commonly manifests as a hepatic mass [3,5,7]. Given the nonspecific nature of symptoms, imaging plays a critical role in the diagnostic work-up and therapeutic decision-making process for patients with suspected iCCA. Frequently used imaging modalities include US, CT, MRI, and fluorodeoxyglucose-positron emission tomography (FDG-PET) [4,8,9]. On US, iCCA typically presents as a homogeneous mass with larger tumors (typically >3 cm) exhibiting hyperechoic characteristics, and smaller tumors exhibiting hypoechoic features. Some cases may also exhibit a hypoechoic halo surrounding the tumor [[9], [10], [11], [12]]. CT depicts iCCA as a non-capsulated, hypodense mass with potential retraction of the hepatic capsule in some patients. Contrast-enhanced scans in the arterial and portal venous phases demonstrate peripheral rim enhancement with progressive centripetal filling. Delayed phase enhancement, which is attributed to fibrous tissue, is a distinctive hallmark of iCCA [5,8,9,12,13]. MRI features of iCCA include indistinct margins, characterized by hypointensity on T1-weighted images and heterogeneous hyperintensity on T2-weighted images. Dynamic images demonstrate peripheral enhancement during the arterial phase, followed by gradual centripetal filling of the tumor. Delayed imaging indicated contrast agent pooling, suggesting fibrosis [5,[8], [9], [10],^14^] (Supplementary material, Fig. S1). FDG-PET is useful for evaluating tumor extent, lymph node metastasis, and distant metastasis, aiding in identifying patients requiring surgical intervention. Elevated tumor marker levels may be useful in distinguishing between different diagnoses; however, the sensitivity of CA19-9 for CCA is approximately 50%–70 % when levels exceed 100 U/mL. The utility of CA19-9 is limited by its tendency to yield false-positive results in cholangitis and other benign biliary tract diseases. Additionally, it should be noted that CA19-9 levels are not detectable in individuals with the Lewis antigen-negative phenotype, which accounts for approximately 7 % of the general population [4,6,7,9,15]. Therefore, the histopathological assessment of biopsy specimens is crucial for the accurate diagnosis of iCCA.

HCE caused by *E. granulosus* infection is prevalent in pastoral regions and is a substantial public health concern worldwide [16]. The initial stages of HCE frequently manifest with subtle symptoms, including decreased appetite and weight loss, thus complicating the diagnosis. Serological tests for echinococcosis exhibit considerable variability in sensitivity and specificity [[16], [17], [18]]; as such, imaging has become the primary diagnostic modality. Based on US imaging findings, the World Health Organization Informal Working Group on Echinococcosis (WHO-IWGE) categorizes HCE into 5 distinct types: CE1 to CE5. Each lesion type exhibits characteristic imaging features, with CE1 lesions manifesting as round, homogeneous cystic lesions in the liver on US, characterized by smooth and intact cystic walls and a bilayer structure. Within the cyst fluid, Taenia scolex floats with changes in posture, exhibiting a classic “snowfall” appearance. Conversely, CE2 cysts exhibit small cystic structures of varying sizes, often displaying “hive” or “cartwheel” configurations as typical cystic alterations. CE3 is identified by the presence of a curled or folded membranous echo within the endocyst wall, commonly referred to as the “ribbon sign”. CE4 presents as a solid lesion with a distinct capsule and internal echoes that exhibit a mixture of layers of varying intensities. In cases in which CE5 is partially calcified, it typically appears as an arc-shaped hyperechoic structure on ultrasound, accompanied by a broad acoustic shadow. The fully calcified cyst walls at CE5 exhibit an eggshell-like hyperechoic pattern on imaging [18,19]. CT imaging commonly reveals the presence of ≥1 round or oval cystic lesions with water density within the liver parenchyma, characterized by distinct borders and a lack of enhancement following contrast administration. The cyst wall is typically visualized as a slightly hyperdense linear band. The characteristic attributes of various subtypes are similar to those observed on US imaging [17,18,20]. MRI typically reveals ≥1 round or oval lesions within the liver parenchyma, which are characterized by smooth, well-defined edges. The cystic fluid appeared hypointense on T1-weighted images and hyperintense on T2-weighted images, with consistent signal intensity. The cyst wall was uniformly thin or thick, with a distinctive low signal on T2-weighted images. The distinguishing characteristics of the various subtypes are comparable with those observed on US imaging. Furthermore, the calcific cyst wall demonstrated hypointensity on both T1- and T2-weighted images [17,20] (Supplementary material, Fig. S2).

The incidence of misdiagnosis of CCA and cystic diseases is uncommon, with only 4 documented cases reported, in which CCA was confused with HCE, each presenting with unique characteristics [[21], [22], [23], [24]]. One such case was reported by Bacher, involving a 76-year-old female with biliary cystadenocarcinoma, which, due to perforation of the bile ducts and invasion into the duodenum, appeared radiologically as a partially calcified, septated tumor with nodular components and multiple air-fluid levels, resembling an infected HCE [21]. Molina documented a case involving a 73-year-old male presenting with symptoms of abdominal pain, fever, and jaundice, with imaging revealing multiple cystic masses suggestive of cholangitis caused by HCE. The patient was initially treated with antibiotics, followed by left hepatic lobectomy after the resolution of cholangitis, with subsequent pathology confirming multicentric cholangiocarcinoma [22]. Serrano reported a case involving a 64-year-old female admitted for acute cholangitis, in whom a well-defined cystic mass, measuring 70 mm, consistent with HCE was incidentally discovered on radiological examination. Left hepatectomy was performed, and subsequent histological analysis confirmed the presence of an intraductal papillary neoplasm of the bile duct [23]. IIn contrast, Mersad Alimoradi reported a case involving a 64-year-old woman who manifested new-onset jaundice and was diagnosed with a Klatskin tumor based on magnetic resonance cholangiopancreatography findings. However, surgical exploration revealed that the obstruction was attributed to rupture of the HCE [24]. Biliary tumors typically do not manifest as cystic masses, which reduces their likelihood of confusion with HCE. However, in some cases involving necrosis, perforation, and other complications, biliary tumors may present as cystic masses. Therefore, it is important to consider these conditions in the differential diagnosis of cystic liver disease.

Multiple factors contributed to misdiagnosis in the present case. First, both iCCA and HCE lack distinctive early symptoms and often present with nonspecific signs. The presence of common symptoms such as upper abdominal pain, nausea, and vomiting, as observed in this instance, are frequently associated with digestive system disorders, making it challenging to differentiate between the 2 conditions [4,17,18]. Second, the patient's residence in Changji, a region with a high prevalence of echinococcosis in Xinjiang, coupled with a previous history of HCE, likely influenced the diagnostic bias toward HCE. Moreover, the occurrence of necrosis in iCCA lesions is uncommon because tumors exhibiting necrosis or mucin production typically exhibit a lack of enhancement in the central region during enhanced scanning and maintain low density throughout all 3 phases. In this particular case, the lesion did not exhibit the typical homogeneous echoic area characteristic of iCCA but instead exhibited heterogeneous internal changes with membranous medium-level echo accumulation, resembling typical US features of HCE (CE4). The CT findings were congruent with this observation because the necrotic lesion exhibited marked enhancement following contrast administration and did not manifest the anticipated delayed-phase enhancement characteristic of iCCA. This aligns more closely with the features of HCE, in which lesions do not enhance post-contrast [3,5,6]. MRI revealed that the lesion exhibited a round and cystic morphology characterized by long T1 and T2 signals, consistent with the typical imaging features of both iCCA and HCE. The internal strip-like signals with slightly shorter T1 and T2 values were interpreted as potentially representing degenerated and necrotic endocytic material, whereas the peripheral ring-like short T2 signals resembled the signal pattern of the cyst wall and aligned more closely with HCE. In conclusion, the patient's symptoms were atypical, the epidemiological findings were aligned with those of HCE, and there was a relevant medical history. The imaging results revealed the unique characteristics of a few lesions with necrotic iCCA, resembling the typical imaging features of HCE and potentially leading to misdiagnosis.

## 4 Conclusion

The imaging characteristics of special iCCA with necrosis differ significantly from those of typical features, potentially resulting in missed diagnoses and misdiagnoses. Consequently, when interpreting imaging data for diagnostic purposes, it is imperative to recognize not only their typical characteristics, but also their unique presentations. This case underscores the importance of considering iCCA with necrosis as a potential differential diagnosis for HCE. Simultaneously, a patient's epidemiological background and medical history can serve as valuable diagnostic indicators. However, they also have the potential to contribute to confirmation bias, thus impairing the accuracy of diagnoses. As such, a holistic approach that considers all relevant factors is essential in the diagnostic process, with an emphasis on recognizing atypical disease manifestations to mitigate the risk for diagnostic errors. We hope that this case of misdiagnosis can serve as a differential diagnostic reference for other clinicians when they are diagnosing similar cases. One limitation of the present study is that it was based on a single case, which can only serve as a reference for clinical experience and cannot provide reliable evidence through statistical analysis.

## Ethics statement

Written informed consent was obtained from the individual(s) for the publication of any potentially identifiable images or data included in this article.

## Funding

10.13039/100009110Natural Science Foundation of Xinjiang Uygur Autonomous Region (2022D01D17).

## Data availability statement

Data associated with the study has never been deposited into any publicly available repository.

## CRediT authorship contribution statement

**Dalong Zhu:** Writing – original draft, Investigation, Data curation. **Abuduhaiwaier Abuduhelili:** Writing – original draft, Investigation, Data curation. **Alimu Tulahong:** Writing – original draft, Investigation. **Chang Liu:** Writing – original draft, Investigation. **Tiemin Jiang:** Writing – original draft, Investigation. **Yingmei Shao:** Writing – original draft. **Tuerganaili Aji:** Writing – review & editing, Funding acquisition, Conceptualization.

## Declaration of competing interest

The authors declare the following financial interests/personal relationships which may be considered as potential competing interests:Tuerganaili Aji reports financial support was provided by 10.13039/100009110Natural Science Foundation of Xinjiang Uygur Autonomous Region. If there are other authors, they declare that they have no known competing financial interests or personal relationships that could have appeared to influence the work reported in this paper.

## Figures and Tables

**FigS1:**
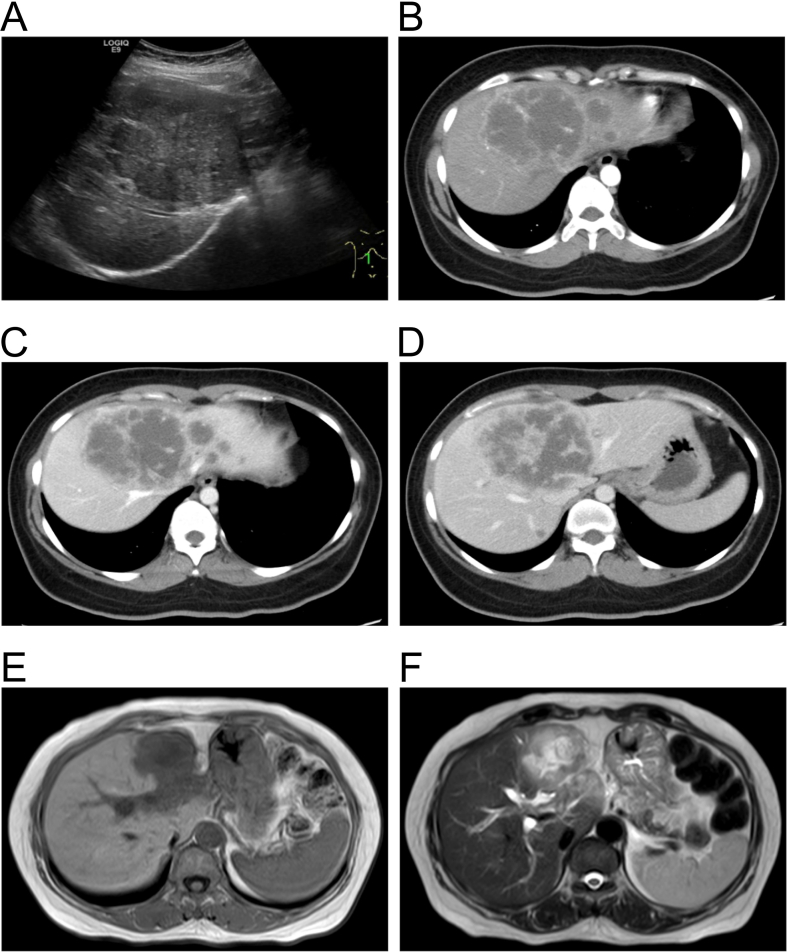
Imaging of intrahepatic cholangiocarcinoma.

**FigS2:**
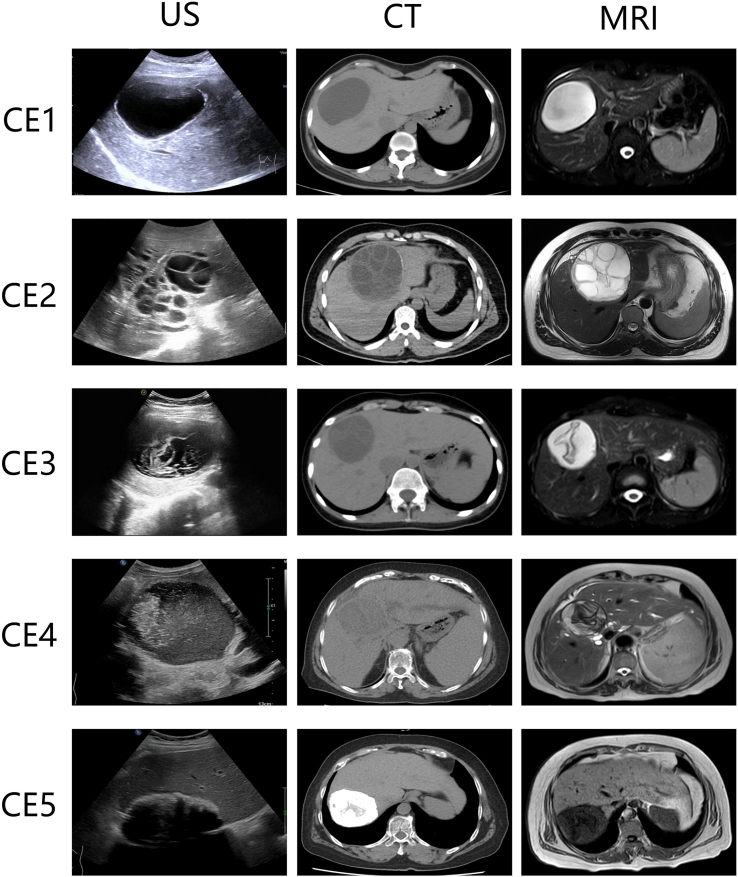
Imaging of hepatic cystic echinococcosis.

## List of abbreviations

iCCA: intrahepatic cholangiocarcinoma
HCC: hepatocellular carcinoma
HCE: hepatic cystic echinococcosis
CCA: cholangiocarcinoma
CA19-9: carbohydrate antigen 19–9
CA50: carbohydrate antigen 50
US: ultrasound
CT: computed tomography
MRI: magnetic resonance imaging
FDG-PET: fluorodeoxyglucose-positron emission tomography
WHO-IWGE: World Health Organization Informal Working Group on Echinococcosis
